# Prediction and Potential Spatially Explicit Spread of COVID-19 in Mexico’s Megacity North Periphery

**DOI:** 10.3390/healthcare8040453

**Published:** 2020-11-02

**Authors:** Maria de la Luz Hernández-Flores, Jair Escobar-Sánchez, Jesús Eduardo Paredes-Zarco, Giorgio Alberto Franyuti Kelly, Lamán Carranza-Ramírez

**Affiliations:** 1 Consejo Ejecutivo del Complejo Científico y Tecnológico Sincrotrón, Boulevard Ciudad del Conocimiento y la Cultura, Manzana 10 Lote 1, Col. Santa Catarina, San Miguel Tornacuxtla, San Agustín Tlaxiaca 42163, Mexico; jairescobar16@hotmail.com (J.E.-S.); lamancarranza@hidalgo.gob.mx (L.C.-R.); 2 INABISA, Investigación Aplicada para el Bienestar Social y Ambiental, Rio Papagayo #10, Ampliación el Palmar, Pachuca 42088, Mexico; jeparedes@ciencias.unam.mx; 3 Medical IMPACT, Dept. of Infectious Diseases, Gutemberg 51, Verónica Anzures, Miguel Hidalgo Ciudad de Mexico 54050, Mexico; giorgio.franyuti@gmail.com

**Keywords:** COVID-19, spatial analysis, density metrics, epidemic, modeling

## Abstract

The novel COVID-19, detected in Wuhan, China, has reached almost every city across the globe, and researchers from many countries have used several epidemiologic models to describe the epidemic trends. In this context, it is also important to know the geographic extent of the infected population. Following this approach, a Gumpertz model was adapted with official data from the state of Hidalgo, Mexico, in order to estimate the people infected during this COVID-19 pandemic. We found, based on the adjusted data, the highest value in infected people according to official and theoretical data. Furthermore, using a geographical analysis based on geostatistical measures related to density of demographic and economic data, traffic level and geolocation, raster files were generated to estimate probability of coronavirus cases occurrence using the areas where the contagion may occur. We also distributed the maximum contagion obtained by the epidemic model, using these raster files, and a regression model to weight factors according their importance. Based on this estimated distribution, we found that most of the infected people were located in the southern border, a trend related to the economic strip in the southern part of Hidalgo State, associated with its vicinity to the Megacity of Mexico.

## 1. Introduction

Since the appearance of the novel coronavirus COVID-19 in December 2019 in Wuhan, the World Health Organization has issued an alert on the “transmissibility, consequences and manifestation of COVID-19 infection” [1]. Since then, due to its rapid spread throughout the world in the first months of 2020, this virus has been the subject of multiple studies, including geographical distribution of infections [2], the most vulnerable areas and the variables [3] that promote greater contagion.

Governments of subregions (states) and nations need to know the contagion dynamics in order to make decisions [4]. They must also know how the occurrence of cases in the territory will be, in order to manage their resources and attend to the emergency according to the geographical conditions that define the territory [5].

Any infectious disease has a distribution component of the susceptible, infected and exposed population related to its density [6]. However, not only the population density is capable of increasing the possibility of contagion. Factors such as the concentration of services and work centers, and the contact caused by public and private transport, have been considered places where contagion mainly occurs.

Although there have been several approaches that attempt to reconcile the possible number of infected with the occurrence of their spatial patterns, as shown in the following table, it is necessary to mention that there are few published approaches for developing countries; this is generally due to the limited availability of data for sub-national levels within the developed countries studies.

Before the COVID-19 outbreak, several studies studied the spread of other diseases: among the most studied cases was the AH1N1 influenza. In this case, we take as background two of the main approaches included in these previous studies: (a) studies where simulations or geostatistical tools were performed to estimate the geographical spread of infectious diseases; (b) assessments of how some socioeconomic factors are related to the presence of infections in a given space. Table 1 describes main studies in both.

Several predictive models have been applied to forecast and describe the tends and outbreak of COVID-19 pandemic. These methods explored the estimation of infected, recovered, or deaths, mainly during this pandemic. The most common methods are explored by using the SIR method and its variants: SEIR or SIR model [15,16]; other methods have reported improved results by using the Gumpertz model or logistic models [17].

The aim of this work is to investigate the mechanics of spread of COVID-19 in a subregional area (Hidalgo State), located in Mexico, focusing on geographical spreading and the relationship between socioeconomic indicators and spreading, by using GIS and statistical tools.

To do so, firstly, the pandemic trend in Hidalgo state was described, defining the most fitting model (1) and then carrying out a geographical approach (2), based on the probability of infection (3) as well as density. Finally, a regression model, with Box-Cox transformation was used (4) in order to identify the main variables which could have any impact on COVID-19 geographical spread.

We found that we were able to use the Gumpertz model, and by using official data, we found a peak on day 136; then, we defined, by using the Montecarlo model, the highest possible probability of each age group and place of contact: we found that the highest values of probability of infection are for adults who attend workplaces. Finally, the assessment of the relationship between the spread of cases and the socio-economic factors showed that population density and the workplaces in each locality are factors that impact COVID-19 spreading.

## 2. Study Case

Developing countries are often more vulnerable to health risks, and the spread of the pandemic can occur in spatial trends related to weak controls that are different to those from developed countries. Additionally, lack of data is a common problem; in this study we explore the spread of COVID-19 as a spatial variable related to the density of demographic factors, defining which are the main factors in a subregional area: The State of Hidalgo, México.

**Figure 1:**
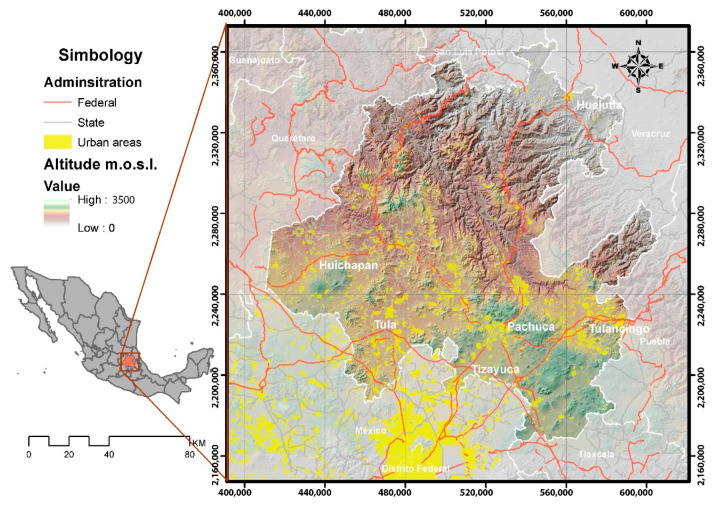
Location of the state of Hidalgo in Central Mexico.

The state of Hidalgo is located in central Mexico, in the northern part of the periphery of Mexico City (Figure 1). The municipalities in the southern part of the state belong to the Megalopolis of Mexico City and, therefore, have a strong dynamic of goods, services, and people mobility towards the center of the city and the rest of peripheral cities such as Puebla, Querétaro, Cuernavaca, Toluca, and Tlaxcala [18]

Due to this high dynamic of interactions, the level of contact is very high, and thence infectious diseases such as the new type of Coronavirus have a high spread from Mexico City to peripheral cities, as other studies that discuss the geographical scope of the epidemics have shown [9].

Since the first cases appeared in Mexico City on 28 February 2020, an attempt was made to establish models that explain the monitoring of the pandemic. However, most of the approaches have been carried out at a federal level, and the geographic definition has been limited to the realization of thematic maps, in some cases including municipalities, which constitute an effort to inform the population but do not provide a greater level of analysis for decision-making, which have been reported in [19].

## 3. Materials and Methods

### 3.1. Materials

To define how COVID-19 would be transmitted, it is necessary to establish a contact network. This network is used to represent how individuals interact and have contact with other people in different places. According to Bian [20], and referred to by Mao and Bian [7], there are four types of places (homes, workplaces, service locations with transportation). To define data for the analysis network, another Latin American case of study was used. Grijalva, C. et al. [21] performed a contact network where the nature of the contacts by age is defined.

Data collection from Table 2 is necessary to define the spaces where these contacts can occur, and the level of contact for each age group and produce the geographical approach of probability, and also to assess how COVID-19 could spread in the State of Hidalgo and how some socioeconomic and demographic factors are related to this spread.

To do this, data were collected from the sources in Table 2.

### 3.2. Methods

To investigate the mechanics of geographical spread of COVID-19 and the relationship between socio-economic indicators and spread, we first described the pandemic trends in the state of Hidalgo, defining the most suitable model (1) and then carrying out a geographic approach (2), based on the probability of infection (3). Finally, a regression model, with Box-Cox transformation, was used (4) in order to identify the main variables which could have any impact on COVID-19 geographical spread.

#### 3.2.1. Estimation of Pandemic Trends

Two models were used to describe the trend of the pandemic in the state of Hidalgo.

(a) SEIR and SEIRS+ model. First, a SEIR model in its standard form requires the parameters of infectious, incubation, and recovery rates. In our first approach with the SEIR model, we considered the parameters 0.626, 0.19, and 0.344, respectively.

After that, we used the SEIRS+ Network Model package that includes an implementation of the Extended SEIRS model in stochastic dynamical networks. In this approach, individuals are represented as nodes in a network, and parameters, contacts, and interventions can be defined according to Ryan, S.E, cited by Hoque, M. E. and Das [27].

When using the SEIRS+ module, we assigned same values, so beta = 0.626, sigma = 0.19, and gamma = 0.344 were used to run the epidemiological model on the demographic network; this demographic network is computed using the proposed python function mentioned above. This includes demographic data from Hidalgo state, such as household size, age of members, and household statistics.

(b) Gumpertz model. The official data were fitted by using the Gumpertz model. This model has a self-regulated growth function, where growth rate decays exponentially, after reaching an inflection point. Gumpertz function is similar to a logistic function, but its less symmetrical nature makes it more suitable for biological phenomena. This is expressed as:

(1) $$C_{a}=ae^{-be^{-ct}}$$

where *a* is the growth and is a maximum asymptote; *b* is a constant adjusted with initial data and defines the function displacement on x. *c* is a constant related to intrinsic growth capability.

Subsequently, the first derivation of Equation (1) was obtained, to be interpreted as the curve that defines new daily cases, as in Equation (2).

(2) $$P_{i}=ae^{-e^{b\left(t-c\right)}}+e$$

Equation (2) allows estimating future covid-19 cases between the following days of pandemic, considering the maximum point of this function as the pandemic peak in Hidalgo.

With the data of new cases in the state of Hidalgo, an adjustment of the behavior of the curve was carried out. The adjustment of the theoretical curve was reviewed, considering that the official data in the initial 150 days were required to define the initial parameters a, b, and c, in order to integrate them into the Python code that was previously made.

#### 3.2.2. Geographical Approach

Most infectious diseases have a direct and positive correlation with population density and other socio-economic factors related to density. Especially COVID-19 has demonstrated this characteristic [8]. For this study, calculations of different densities were made: (a) of total population; (b) of housing (c) and workplaces, (d) which are service locations; (e) of age groups such as children (0 to 14 years), economically active population (15 to 59 years), and older adults (60 years and older).

To calculate these densities, we used the Kernel algorithm, which calculates a magnitude per unit area from point or polyline entities using a Kernel function to fit a smoothly tapered surface to each point or polyline.

**Figure 2:**
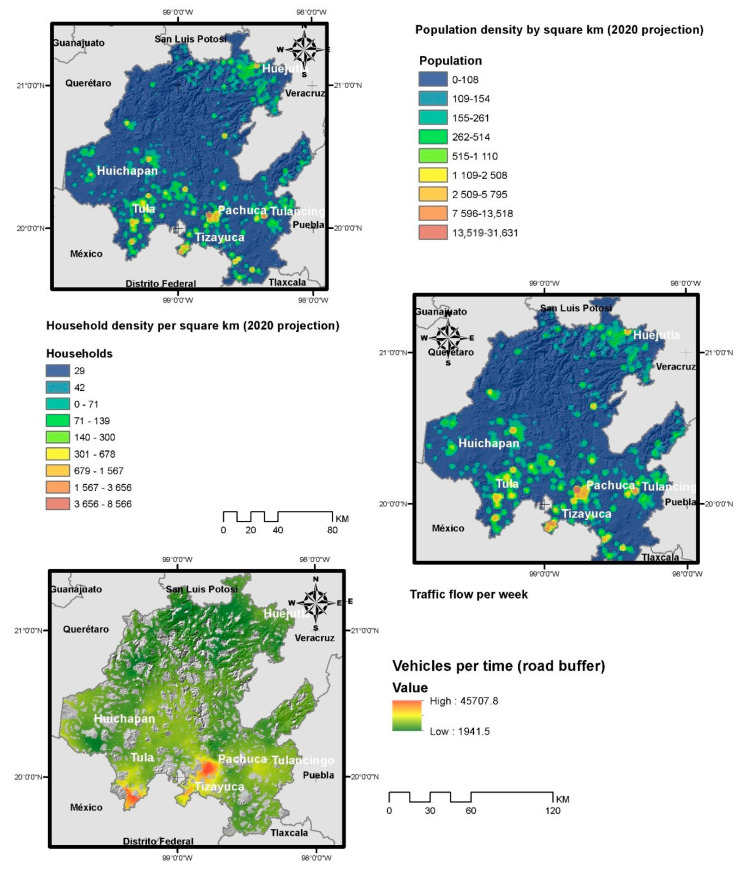
Geographical description of the variables related to density and that affect the level of contagion.

We considered 3 age groups and 4 contact areas mentioned in the previous paragraphs. According to the network of contacts defined by Bian [3], we geographically referred these spaces through the ArcGis software using the following method:

(a) Workplaces: The economic units defined in the DENUE national directory of economic units [23] were assigned to each locality through a proximity analysis that assigned each workplace to the closest locality.

(b) Places of service provision: The economic units of service provision (sectors 41 to 95) defined in the DENUE [23] were assigned to each locality through a proximity analysis assigned to each service provision site.

(c) Housing in each locality: We projected the growth of housing until 2020 based on the growth rate between 2010 and 2015.

(d) Traffic level: Capacity level of the main state highways, during 2018. In order to determine the level of capacity in the localities, the Kriging method was interpolated and a road buffer was established, according to Figure 2.

Representation of population densities and counts based on the kernel algorithm shows that the concentration of households, population, work centers, service supplies centers, and traffic levels denoted the impact of main population centers and metropolitan areas. Some of these factors are represented in Figure 2. In addition, a database obtained collects information on the number of people in each age range, the average population density of each locality, and the number of economic units in each sector.

#### 3.2.3. Probability of Infection

We estimated the probability of infection in the State of Hidalgo. According to Mao and Bian [7], the proportion of infections in a pandemic scenario in households is in a range of 47–51%, while at workplaces it is estimated at 37–42%, and for service places, 11–12%; we consider these values as infection rate per place with a uniform probability distribution. On the other hand, Grijalva et al. [21] estimate the proportion of contacts by category for contact duration and age ranges. This contact proportion has been interpreted as the *E_contact_* variable with a probability distribution of contacts per age; as age increases so does the duration of contact. The maximum and minimum values of *E_contact_* range from 0 to 1.

To obtain the infection rate per age *I_Gedad_*, the average of the minimum and maximum value of infection rate per place and proportion of contact per age were calculated for each type of place (households, workplaces, and service places). Those maximum and minimum values were estimated by using Oracle Crystal Ball utility.

Once obtained, the *E_contact_* and *I_Gedad_* values and the infection probability per age and place were calculated. For this, Monte-Carlo simulations were carried out considering the maximum and minimum values as well as their probability distributions.

The reception of infection through a contact was simulated based on the probability of infection:

(3) $$p=E_{contact}\times I_{Gedad}$$

where *E_contact_* is the effectiveness of a contact for infection. In this case, we consider close contacts. *I_Gedad_* is the age-specific infection rate and is expressed between 0 and 1. The probability *p* can be estimated for *I_Gedad_* and *E_contact_*. Contact intensity was weighted between 0 and 1 in the defined areas: work, services, and households, with data from the contact network developed by Grijalva et al. [21].

In this way, infection probability was defined by each age group and by place of contact. As described by Hamidi, infection probability is highly related to density [14], so maps describing probability weighted by density were estimated. Additionally, a consideration on mobility restrictions of 30% (average) according to analytics by Google was applied to contact factors in workplaces and transportation.

#### 3.2.4. Relationship Between Socio-Economic Indicators and the Spread of COVID-19 Defined by Map Algebra

Initially, each case was plotted as a point per locality. As described above, densities were estimated to population, households, workplaces, and service supply places using the kernel algorithm, as well as traffic level by krigging. Also, variables such as latitude, longitude, and altitude are implicit in raster files. Similar approaches were used by Copiello and Grillenzoni [6] and Hamibi, S. et al. [14].

We used the least squares method defining the function that describes the spread in the territory in order to achieve an accurate estimation of spatially explicit cases. The dependent variables tested such as latitude, longitude, and altitude were extracted from raster files, and the kernel algorithm was used to obtain population density, households, work places, places of service provision, and traffic flow level. Independent variables were tested as the number of cumulative cases.

A BoxCox transformation was applied to the original model correcting specification assumption of autocorrelation and normality, which were evaluated with Durbin–Watson and Kolmogorov–Smirnov tests. Significant variables were used to address the geographic distribution of cases.

The study used Map algebra to distribute Covid-19 cases along the state of Hidalgo, based on results of BoxCox regression, weighting variables according to their coefficients as well as their significant values. Kernel distributions rasters were introduced as factors to estimate the distribution of COVID cases; this way, the resulting raster is more realistic than the simple kernel distribution cases.

## 4. Results

### 4.1. Trend of Pandemic in the State of Hidalgo

With the growth model of the state of Hidalgo data, and the following adjustment data: a = 19,370, as the maximum number of estimated cases for each day; b is the estimated growth rate estimated for total COVID-19 cases, so b = 0.1426, according to the Gumpertz; c = 136.6 is the days on which the number of new cases per day can occur. Using the difference of squares, the adjusted measure result was 0.997. As indicated above, the SEIR approach was useful in the early days of the pandemic, but the function became excessive, and furthermore its geometric nature does not explain the behavior of pandemic.

In the SEIR model initially considered, a high number of accumulated cases was predicted (around 7% of the total population, which means more than 200,000 cases); meanwhile, the maximum Gompertz curve was forecast at 17,000 cumulative official cases. That is why we consider the SEIR as difficult to validate with the test rate in México (0.4–11 tests per thousand persons), while we were able to validate the Gumpertz trends with official data. As well as Gumpertz, the results can be validated, at least with official data.

**Figure 3:**
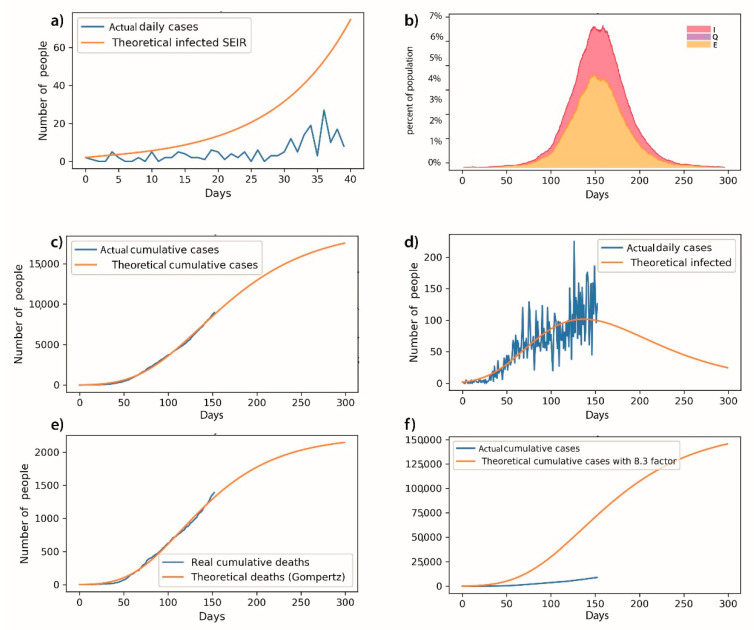
The adjustment of the SEIR, SEIRS+ package, and Gumpertz model is observed with the parameters described above. The resulting curves are shown below, where curve (**a**) shows the initial adjustment of SEIR curve, (**b**) shows the adjustment of SEIRS+ Network Model curve, (**c**) shows the infection curve with daily official cases, (**d**) shows the adjustment of official data with the theoretical curve development, (**e**) shows the cumulative death curve, and (**f**) shows the proportion of theoretical deaths according to the model and with the expansion factor (8.3) defined by the Federal Ministry of Health on 4 April 2020 [28] and described by Torrealba et al. [17].

To validate the results obtained, data of the next 20 days were analyzed for validation of the Gompertz model, from which an inference was obtained that has a maximum error of 4% of the official data. Figure 3 displays our results (see Appendix A).

### 4.2. Representation of the Areas Where There Is a Greater Probability of Contagion

By applying the Montecarlo model with the purpose of obtaining the most possible probability ranges for each population group and contact place. We found that for the adult population group with the highest infection probability, the ranges were 0.06 to 0.26 for workplaces, 0.07–0.27 for service places, and 0.06–0.27 for households. The seniors group showed a probability of infection of 0.06 to 0.24 in service places, and 0.05 to 0.23 for workplaces and households. Finally, the children group showed probability ranges from 0.05 to 0.24 for workplaces (including schools) and service places and 0.05 to 0.23 in households.

**Figure 4:**
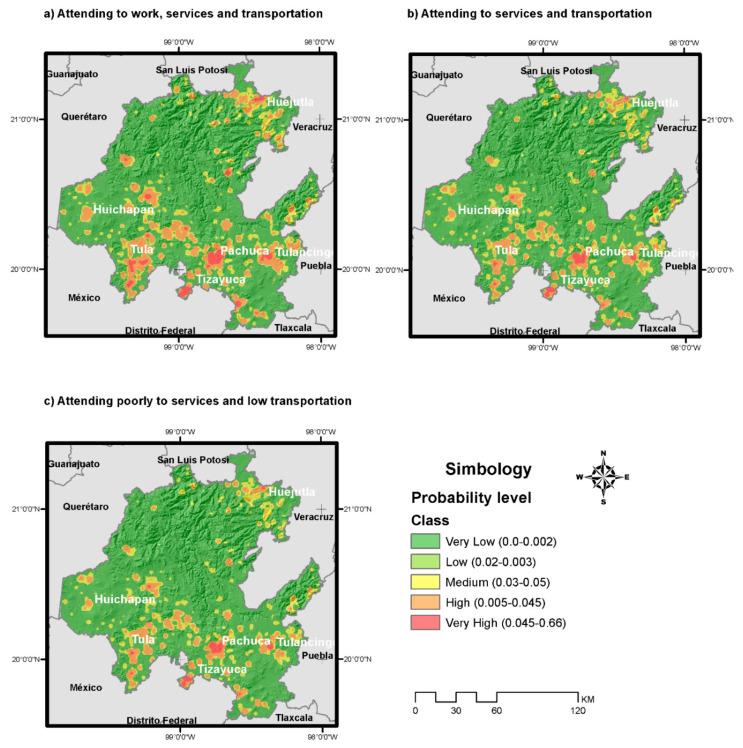
Probability of infection weighted by population density, considering (**a**) no restrictions in mobility, (**b**) 30% restrictions in mobility to services, and (**c**) more than 30% restrictions in mobility.

To represent these probabilities in maps, Figure 4 was obtained. In each part of the figure, different assumptions about mobility restrictions were considered. The first part does not consider any mobility restriction, while part (b) and (c) do.

Regarding Figure 4, in part (a), the infection probability without any restriction measures, where people attend to work, and use services and public transport according to their geographical location, was determined. In part (b), infection probability was estimated considering a restriction in mobility to services by decreasing 30% in transportation. In part (c) of the figure, infection probability was estimated considering a higher percentage in mobility due to restrictions (more than 35%).

As shown in all cases in Figure 4, metropolitan areas of the southern fringe of Hidalgo have the highest values; this can be explained by the proximity to Mexico City and its interactions.

### 4.3. Relationship Between Socio-Economic Indicators and the spread of COVID-19 on Map Algebra

**Figure 5:**
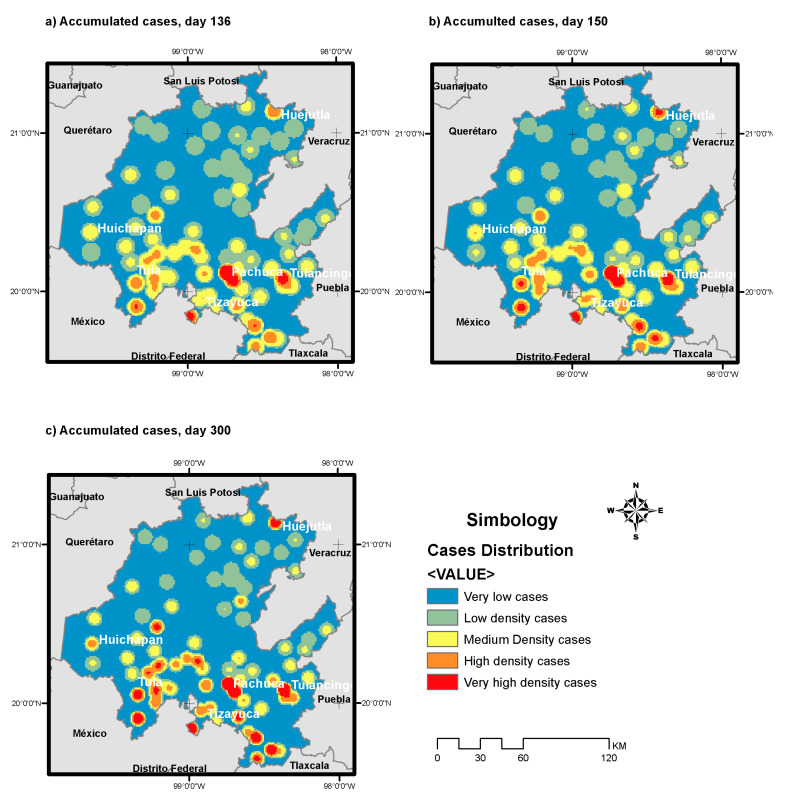
Case distribution based on population and workplaces density, based on kernel. (**a**) Accumulated cases at day 136. (**b**) Accumulated cases at day 150. (**c**) Accumulated cases at day 300.

With these last variables, which are based on territorial density, a distribution model was established, based on a regression with BoxCox transformation. This model allows determining the main factors that define COVID-19 cases throughout the territory of the state of Hidalgo, according to the previously modeled Gompertz curve cases and their geographical distribution. COVID-19 cumulative cases were distributed. The expression that describes this approach is given by:

(4) $$C_{a}=1.11+0.05P^{*}+0.0001W^{*}+e$$

where *C_a_* means the accumulated cases, 1.11 is the intercept, *P* is the population density distributed by kernel algorithm, and *W* is the density of workplaces per square kilometer, also obtained by kernel. The coefficient of determination *R^2^* result was 0.543, and both dependent variables were significant at 95% (and are marked by the symbol *), as well as the model. Figure 5 shows the geographical distribution of cases, based on Equation (4), at three different times of the pandemic: day 136, peak on day 150 and day 300.

## 5. Discussion

Regarding the performance of the Gompertz model, although several theoretical curves have been generated, the one presented in this exercise adjusts to what has happened according to reports from the Federal Ministry of Health and the state of Hidalgo. In the same way, it is considered a factor of unknown cases, that according to federal data is set to be 8.3. Hence, cases can officially increase up to 17,000 official cases in 300 days, with 140,000 more unofficial cases considered in the same period.

The low rate of testing in Mexico is still a limitation to more accurate approaches and validations, as according to OECD and the site in our world in data, from Oxford University, testing rates have increased from April to September 2020: in April, 0.4 tests per 1000 people were registered, while in September, 11.11 tests per 1000 people were registered.

Although there are several studies for this purpose, the present work suggests an approach to subregional areas from developing countries with limited data, where, by using not so complex geoprocessing methods, we can obtain valuable information for planning and decision-making during the pandemic. This work describes an estimation of cases based in common models, and describes how age groups’ probability of infection differs according the place where contacts occur; these infection probabilities were spatially distributed by using raster data of population densities, and finally, the raster files produced were used to define by a regression the most significant variables that affect the spread of COVID. Those methods are commonly used in similar studies, but have not been integrated to configure a useful work for pandemic management in subregional areas.

Regarding the obtained results, considering the expansion factor of 8.3 obtained by the epidemiologic analysts of the National Health Council, unofficial cases can be more than 140 thousand cases until day 300 of the pandemic. Previous estimates were performed by using SIR-based studies (2), and also Gumpertz, Logistic function, and neural network approaches, by Torrealba-Rodríguez et al. [17], where Gompertz was found to be an accurate model to estimate COVID-19 cases across the country, and it was the closest data to the validation date, while the logistic model was no so accurate. Additionally, in this work, we only report a difference in squares, and not R^2^, because it is an inadequate measure for nonlinear models [29]. However, SIR-based approaches have also predicted the pandemic.

The probability of infection showed higher values in the adult group in workplaces, which corroborates the results reported in the data by the Federal Ministry of Health [25] and according to maps, the southern fringe is the most affected area in the State of Hidalgo, due to its interaction with Mexico City. In this work, variables such as population density and presence of workplaces were also found to greatly impact the spread of COVID-19, as described by Mameulnd, S.E. et al. [13], Copiello and Grillenzoni [6], and Hamidi et al. [14].

The public policies about pandemic management are still incomplete; although isolation measures have been applied in many countries and their regions, more approaches are needed to improve public health policies in the context of this pandemic.

## 6. Conclusions

Regarding the lack of data for developing countries, logistic efforts can be avoided by using geostatistical data and models, which are tools for decision makers when resources are not sufficient to deal with this pandemic or other disease outbreaks.

According to the models, although a first peak has been reached, cumulative cases are still occurring even 400 days after the pandemic. The probability of infection showed ranges from 0.04–0.23 to 0.06–0.26 with a 90% confidence level; the adult group in workplaces has the highest values.

Population densities, household, workplaces, services supply, and traffic levels are important, but according to the BoxCox regression performed, population density, work places density, and traffic levels are the most important variables, although the last one was on the limit of significance. Those factors can explain COVID-19 case distribution with R^2^ = 0.543. This accentuates the impact of workplaces on the pandemic spread in the State of Hidalgo. These data can guide pandemic management policies in this place.

The isolation policies can be more effective when people are not attending work and can decrease the pressure on supply service centers. It is also necessary to decrease traffic levels as much as possible; this is a challenge because economic activation is necessary. More specific metrics in the state of Hidalgo would be helpful for this kind of research—for example, travel surveys.

The research was based on several tools such as spatial analysis, map algebra, and modeling, adjusted to several models; these approaches are critical to tackle the pandemic challenge in subregional areas of developing countries.

## Figures and Tables

**Table 1:** Spatial approximations for the COVID-19 and other diseases spread.

Author	Case Study	Approaches
Mao and Bian, 2010 ^a^ [7]	Buffalo Metropolitan Area and Niagara Falls	An individual spatially explicit model is established to replicate a network of urban contacts and simulate influenza epidemics. The resulting epidemic curves and infection intensity maps are used to analyze transmission dynamics.
Liang Mao, 2014 ^a^ [8]	Applicable to any city with 1 million inhabitants.	It proposes a spatially explicit agent-based model to simulate a triple diffusion process in a metropolitan area of 1 million people.
S. Zhao, 2020 ^b^ [9]	Mainland China	The association between Wuhan’s domestic passenger load and the number of confirmed 2019-nCoV cases in different cities in China is examined and explored.
Desjardins, M.R., (2020) ^b^ [10]	United States	A foresight space-time analysis detecting statistically significant space-time clusters of COVID-19 at a federal level in USA is conducted.
Kang, D. (2020) ^b^ [11]	Mainland China	This study explored the spatial epidemic dynamics of COVID-19 in mainland China. The Moran I Spatial Statistic with various neighbor definitions was used to perform a test to determine if there was a spatial association of COVID-19 infections.
Botá, A. et al., 2020 ^a,b^ [12]	Sweden	The Generalized Inverse Infection Method (GIIM) is performed to identify socioeconomic, travel, and environmental factors contributing to the spread of H1N1 in Sweden.
Mameulnd, S.E. et al., 2019 ^b^ [13]	Review to several cases in many countries, mainly in Europe.	A systematic review and meta-analysis on the link between socioeconomic status and pandemic outcome are carried out.
Rader B. et al., 2020 ^b^ [2]	China	Spatial variables for cities in China are analyzed alongside case count data to investigate the role of climate, urbanization, and variation in interventions across the country.
Copiello and Grillenzoni, 2020 ^b^ [6]	China	The relationship between demographic, socio-economic, and environmental conditions and the spread of the novel coronavirus COVID-19 in China is analyzed with spatial regression models
Hamidi et al., 2020 ^b^ [14]	USA	Using SEM analysis, the relationship between county density and COVID-19 mortality and infection rates is investigated.

^a^ Simulations or geostatistical tools to estimate the geographic spread of infectious diseases; ^b^ assessments of how some socioeconomic factors are related to the presence of infections in a given space.

**Table 2:** Information sources for the study of the State of Hidalgo.

Data	Data Type	Source
Polygons of localities of Hidalgo (INEGI)	DB * and geo-referenced polygons	Directorio estadístico nacional de unidades económicas 2015 [22]
Population characteristics by locality - Housing by locality - Total population - Population from 0–14 years - Population from 15 to 59 - Population from 59 and over	DB	Directorio estadístico nacional de unidades económicas [23].
Working Centers	DB and geo-referenced polygons	Directorio estadístico nacional de unidades económicas [23].
Service Centers	DB and geo-referenced polygons	DENUE [23]
Average vehicle capacity	DB	Secretaria de comunicaciones y transportes. [24]
Cumulative positive cases of COVID, officially detected	DB	Secretaría de Salud de Hidalgo. [25]
SEIR model data for Mexico City	Parameter values	Gobierno de la Ciudad de México [26]

* DB: Database.

## Appendix A

Databases, raster files and codes in R and python are available at: https://github.com/JairEsc/Spatially-explicit-potential-transmission-of-COVID-19-in-Hidalgo.
